# The Role of Kappa‐Opioid Receptor/Dynorphin System in Modulating Neural‐Circuits of Depression and Anxiety—A Review

**DOI:** 10.1111/jcmm.71392

**Published:** 2026-10-06

**Authors:** Cristian Ciotei, Horia Păunescu, Cristina Lungu, Adriana‐Alexandra Marica, Mihai Florin Tănase, Ruxandra‐Cristina Marin, Mihnea Costescu, Ion Fulga, Oana Andreia Coman

**Affiliations:** ^1^ Faculty of Medicine, Pharmacology, Clinical Pharmacology and Pharmacotherapy Discipline “Carol Davila” University of Medicine and Pharmacy Bucharest Romania

**Keywords:** anxiety disorders, kappa‐opioid receptors, major depression disorder, neural circuits

## Abstract

Major depression and anxiety disorders are psychiatric disorders that involve a great number of people. There is a growing need for new and better pharmacological treatments for these diseases. Kappa‐opioid receptor (KOR) antagonists seem to be a promising new pathway for the development of new antidepressants and anxiolytics, but a great challenge is understanding how the various neural circuits work and to what degree they are influenced by KOR modulators. This narrative review is focussed on two main circuits: the Hippocampus‐Amygdala‐Prefrontal Cortex and the Ventral Tegmental Area‐Nucleus Accumbens. The relevant neuroanatomy, their role in modulating behaviour and how KOR modulators can alleviate these diseases are discussed. Concluding, a more holistic understanding of the circuits influenced by the KOR/Dyn system is relevant for developing new therapies. In non‐clinical pharmacology addressed to the KOR/Dyn system, variables such as age, sex and social hierarchy rank can influence experimental outcomes.

**Abbreviations**
ACC: anterior cingulate cortex
AD: anxiety disorders
Amg: amygdala
BA: basal nucleus of amygdala
BDNF: brain‐derived neurotrophic factor
BLA: basolateral amygdala
BMA: basomedial amygdala
BNST: bed nucleus of stria terminalis
CA: cornu ammonis
CeA: central nucleus of amygdala
CREB: cAMP response element‐binding protein
CRF 1‐R: Corticotropin‐Releasing Factor Receptor type 1
CRF: corticotrophin‐releasing factor
D1‐MSN/D2: MSN Medium Spiny Neurons expressing D1/D2 dopamine receptors
DA: Dopamine
DG: dentate gyrus
dlPFC: dorsolateral prefrontal cortex
DOR: Delta‐Opioid Receptor
Dyn: dynorphine
ERK: extracellular signal‐regulated kinase
fMRI: functional magnetic resonance imaging
FST: forced swimming tests
GABA: Gamma‐Aminobutyric Acid
HPC: hippocampus
HT: Hypothalamus
IL: infralimbic cortex
K.O: Knockout
KOR: kappa‐opioid receptor
LC: Locus Coeruleus
LDT: laterodorsal tegmentum
LHb: lateral habenula
MAPK: Mitogen‐Activated Protein Kinase
MDD: major depression disorder
MOR: mu‐opioid receptor
mPFC: medial prefrontal cortex
MSN: medium spiny neurons
Nac: nucleus accumbens
NE: Norepinephrine
NO: Nitric Oxide
Nor‐BNI: norbinaltorphimine
pDyn: pro‐dynorphin
PET: Positron Emission Tomography
PFC: prefrontal cortex
PL: prelimbic cortex
PV+: parvalbumine‐positive
SST+: somatostatin‐positive
tVTA: tail of ventral tegmental area
vHPC: ventral hippocampus
VTA: ventral tegmental area

## 1 Introduction

Major depression disorder (MDD) and anxiety disorders (AD) are issues which a significant number of individuals from teens to the elderly are confronting with [1, 2, 3]. These diseases decrease the quality of life, create vulnerabilities and increase socio‐economic burden. Hence, there is an increasing need to discover new ways of addressing these issues or to repurpose already existing drugs [4, 5].

The kappa‐opioid‐receptor‐dynorphine (KOR/Dyn) system was highly addressed during the last decades, with KOR/Dyn systems being involved in addiction, dysphoria, anxiety, fear behaviour, emotional aspects of pain, negative motivation and many other negative aspects of emotional behaviours [6, 7, 8]. Clinical trials seem promising regarding the usage of KOR‐antagonists as possible antidepressants [9, 10]. This is especially exciting since, unlike the other antidepressants which were first observed to be useful in treating MDD, KOR‐antagonists were also used based on knowledge of the role of KOR/Dyn systems in mediating negative affectivity. It is known that the mu‐opioid receptor (MOR) stimulation is responsible for the addictive properties of opioids while KOR stimulation is responsible for the dysphoric effects, a sort of counterbalance to the MOR system [8]. There is already a large body of literature documenting the anxiolytic and antidepressant effect of KOR‐antagonists such as Norbinaltorphimine (nor‐BNI) and JDTic and the negative effects induced by KOR stimulation [11, 12, 13]. Being a bivalent morphinan dimer, each substituted with a cyclopropylmethyl group on the nitrogen, which is what gives nor‐BNI its high selectivity and long duration of action at the kappa‐opioid receptor.

This simple image of KOR/Dyn's role, while promising, does not satisfactorily represent the reality. In fact, there are some circumstances in which KOR stimulation can have rewarding effects [14] and abolition of all KOR throughout the central nervous system (CNS) is not associated with anxiolytic‐like effects [15]. One must consider that KOR activation is associated with multiple downstream molecular pathways with different outcomes [16] and that different ligands might preferentially activate or not one of these pathways [17]. Furthermore, while generally KOR is coupled with Gi proteins and leads to hyperpolarization, there are situations where KOR activates Gs proteins [18] and instead of hyperpolarization, leads to depolarisation [19]. On the other hand, one has to consider the effects of KOR/Dyn systems at the level of neural circuits, meaning the brain regions where KOR is distributed and the subtypes of neurons on which they are expressed. The part of the neuron on which KORs are represented. Also, the specific behavioural involvement of these circuits should be taken into consideration. Thus, it becomes evident that a far more complicated problem must be unravelled if we want to better understand how to manipulate the KOR/Dyn systems for therapeutic purposes.

This article aimed to highlight the complexity of KOR/Dyn systems in modulating two important brain circuit: the Prefrontal Cortex‐Hippocampus‐Amygdala (PFC‐HPC‐Amg)—and the Ventral Tegmental Area—Nucleus Accumbens (VTA‐Nac). These circuits are involved in reward, fear and anxiety, and as such are important targets for MDD and AD management. This information is necessary in order to guide the researcher in the neuroanatomy, physiology, pathophysiology and pharmacological modulation involved in these diseases of the CNS. In this analysis, the focus consists of describing the involvement of the opioid system, especially KOR/Dyn systems, in the complex pathogeny of MDD and AD.

## 2 Neuroanatomy of the Circuits Involved in the Thymic State or Affectivity in Mice, Rats, Humans and Other Primates

There are two circuits that will be analysed:

Circuit 1: Prefrontal Cortex‐Hippocampus‐Amygdala.

Circuit 2: Ventral Tegmental Area—Nucleus accumbens.

### 2.1 Prefrontal Cortex‐ Hippocampus‐Amygdala

The PFC‐HPC‐Amg‐ circuit is very important for anxiety and fear (see Figure 1.) This section will explore in more detail the functions of this circuit and its individual components.

**FIGURE 1:**
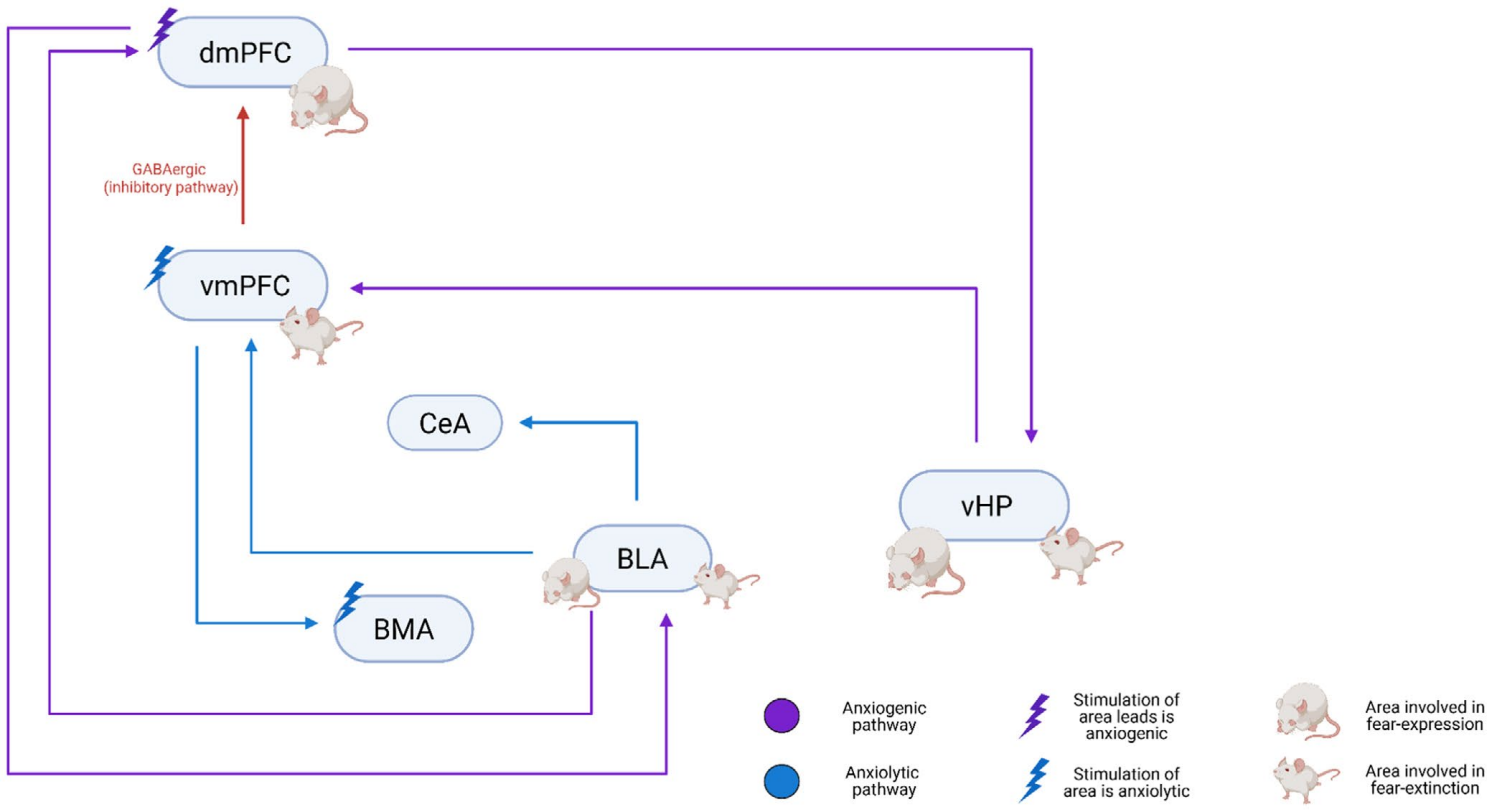
Representation of Prefrontal—Hippocampus—Amygdala circuit involved in anxiety modulation and fear memory; BLA, basolateral amygdala; BMA, basomedial amygdala; CeA, central amygdala; dmPFC, dorsomedial prefrontal cortex; vmPFC, ventromedial prefrontal cortex; vHPC, ventral hippocampus; created in BioRender. Ciotei, C. (2026) https://BioRender.com/q5a66dh.

#### 2.1.1 Pfc

The primate PFC, and especially the human counterpart, is significantly different and more complex than the rodent equivalent, which lacks the more defined regional subdivision found in primates. The existence of a rodent PFC equivalent is a matter of discussion [20]. From a cytoarchitectonic point of view, the rodent PFC is similar to the primate's anterior cingulate cortex (ACC), but electrophysiological and functional a combination of ACC and a less sophisticated dorsolateralprefrontal cortex (dlPFC) [21] can be observed. Also, the rodent's PFC is not as diffusely interconnected as the primate dlPFC [22]. The problem is complicated by the lack of standardized nomenclature in research focusing on rodents and that focusing on humans or non‐human primates [23].

##### 2.1.1.1 In Rodents

The rodent medial prefrontal cortex (mPFC) is comprised of ACC, the prelimbic (PL) or dorsal mPFC and the infralimbic (IL) or ventral mPFC [24] and it sends and receives projections from multiple brain regions such as the amygdala, striatum, ventral hippocampus (vHPC), thalamus, periaqueductal grey, VTA and other cortical regions. As stated above, the rodent mPFC is agranular and it lacks layer IV; instead, the incoming information is sent across all the other layers, and also the output from the mPFC is sent by projections coming from layers II to VI [24].

The PL and IL of rodents have very distinct connectivity patterns [25] which is shown in the manner in which the two areas process anxiety‐related information. PL is bidirectionally communicating with the basolateral amygdala (BLA). PL‐BLA pathway stimulation leads to increased anxiety and induced aversive behaviour in a place avoidance paradigm while its inhibition has anxiolytic effects [26]. In the same time BLA‐PL activity is increased by stress and is also associated with increased anxiety [27].

Contrasting with PL to amygdala connection, IL is sending its output to the basomedial amygdala (BMA) and activation of this circuit but not of the entire IL has anxiolytic effects [28], suggesting that IL is also involved in circuits of anxiety. Furthermore, IL is important also in reducing fear‐expression and consolidating fear‐extinction after fear‐conditioning [29] and inhibiting it affects extinction [30] while stimulating PL leads to increased fear‐expression and prevents the extinction of conditioned fear [31] and its inhibition limits fear expression [30].

##### 2.1.1.2 In Humans

The prefrontal cortex (PFC) is the non‐motor part of the frontal lobe, involved in processing information relevant for higher cognitive functions. In humans, it is roughly subdivided into the medial, lateral and orbital parts [32]. The lateral part is crucial for speech, reasoning and executive functions requiring control of attention, working memory and integration of temporal aspects for accomplishing complex tasks. The medial part is relevant in value‐attribution, salience and motivation; it processes the affective components that offer the reasons for seeking goal pursuit and goal completion [33]. While the orbitofrontal, together with the lateral part, is involved in impulsivity and behaviour control, being relevant for value reassignment and regulating strategy changes, inhibiting an existing behaviour that no longer serves to the purpose and allowing the emergence of a new behaviour [33, 34].

#### 2.1.2 Hippocampus

It can be roughly divided into the dentate gyrus, cornu ammonis (subdivided into CA1, CA2, CA3 and CA4) and the subiculum. Its main input area is the entorhinal cortex which projects mostly through the perforate pathway to the dentate gyrus (DG) region, the projection follows predominantly the classic trisynaptic loop: DG → CA3 → CA1 → entorhinal cortex → DG [35, 36] (for a more thorough discussion on hippocampal anatomy and function see [37]). Apart from this classic processing loop, the CA1 directly sends projections to other, extrahippocampal regions such as different areas of the mPFC and BLA [38] and to the nucleus accumbens (NAc) [39].

The hippocampus is part of the limbic system and it plays a pivotal role in temporal and spatial memory, as well as learning [40].

##### 2.1.2.1 In Rodents

Taking into account the divisions of the rodent's hippocampus, the dorsal part is more involved with spatial representation and episodic memory while the ventral hippocampus (vHPC) plays a bigger role in thymic states processing [41].

vHPC involvement in anxiety‐like states is complex, while vHPC to mPFC activation produces anxiety‐like states, stimulation of vHPC neurons projecting to the lateral septum is anxiolytic [42]. The hippocampus is also important in fear‐conditioning, being relevant for fear‐expression as well as fear‐extinction [30]. Apart from its role in learned‐fear, the vHPC‐mPFC is involved in innate fear, inhibition of this pathway leading to a reduction in avoidance behaviour as well as the loss of the avoidance value representation in mPFC units [43].

Rodents exposed to acute and chronic stress suffer a reduction in the volume of the hippocampus [44]. Also, a dysfunctional vHPC‐NAc pathway and fewer excitatory projections from the vHPC to the cholinergic neurons of NAc were found in a calcium‐binding protein p11 k.o mouse model, a model of depression [39].

##### 2.1.2.2 In Humans

Individuals suffering from major depressive disorder are reported to have decreased hippocampal volume compared to healthy control subjects [45]. Furthermore, the hippocampus is involved in suppressing corticotrophin‐releasing factor (CRF) from the hypothalamus, thus a dysfunctional hippocampus might not properly regulate the corticoid‐stress axis and in turn lead to anxiety and an ever‐lasting sensation of being in danger [46].

#### 2.1.3 Amygdala

##### 2.1.3.1 In Rodents and in Humans

The amygdala is a structure located deep in the temporal lobe comprised of a cortico‐medial region, associated with olfactory information and a basolateral region, phylogenetically newer, associated with the neocortex. The first region is composed of central, medial and cortical nuclei while the latter is formed by the basal, lateral and accessory basal nuclei [47]. It regulates emotional responses, involuntary control, motivation, fear‐memory. The best understood role for emotion processing is that of the basolateral complex (BLA) and the central nucleus of amygdala (CeA) [48] which is further subdivided into a capsular, lateral and medial subdivisions, being important for fear learning, threat conditioning and anxiety‐like behaviours [49]. The bodies of glutamatergic neurons projecting from the basal nucleus (BA) to the mPFC are not anatomically segregated but they form distinct long‐range projections to the mPFC. These neurons are active during fear‐expression after fear‐conditioning and specific inhibition of this pathway during extinction training leads to enhanced memory‐extinction. While neurons projecting to the IL are involved with fear‐extinction and inhibiting these projections during fear‐extinction training causes poorer extinction results [50].

The lateral nucleus is the main input area of the amygdala receiving information from sensory cortices and the hippocampus among other regions, while CeA is the main output region, sending projections to the thalamus and different modulatory areas of the brain stem leading to behaviours such as freezing [47]. Other nuclei also play an important role in receiving as well as sending information; for example, the basal nucleus receives information from the hippocampus and the entorhinal cortex, while the medial nucleus receives projections from the olfactory bulb. A general schema of the information flow involves signals from the lateral nucleus being driven through the basal nucleus and the clusters of intercalated neurons and reaching the CeA [47]. BLA neurons that project to CeA are anxiolytic, most probably by activating inhibitory neurons from the lateral part of CeA which project and hyperpolarise CeA neurons of the medial aspect [51].

The intercalated neurons are found clustered in the white matter between the BLA and CeA. They are mainly inhibitory neurons that are broadly and densely interconnected with each other and which connect the different nuclei of the amygdala. The main cluster, apart from receiving information from other clusters of intercalated neurons and amygdalar nuclei, also receives information from the hippocampus [52]. Furthermore, there are studies supporting the existence of PL projections to the intercalated clusters which are involved in anxiolysis [28]. Present at the level of the medial and main clusters, there are neurons that respond to conditioned fear, which are predominantly found at the level of the medial cluster, and neurons that respond to extinction, which are present in the main cluster [53]. These clusters send reciprocal inhibitory projections and also projections to other regions; for example, the main cluster inhibits BLA principal neurons going to the PL and those of the medial to those BLA neurons sending projections to the IL. Thus, the interplay between the two clusters determines how strongly a neutral stimulus is associated with a threat.

At the level of CeA the information is firstly processed by the lateral subdivision of the CeA before being sent to the medial part and output to extra‐amygdalar targets. The lateral part of CeA presents two important types of inhibitory neuron subtypes that are either activated or inhibited by the conditioned stimulus in a fear‐conditioning setting, being involved in the process of fear‐memory formation and fear‐expression [48].

Amygdala Dyn/KOR system constitutes a system known to contribute to chronic pain and fear‐related behaviour [54]. In humans, the amygdala of individuals having MDD shows an important decrease in pro‐dynorphin (pDyn) mRNA compared to controls, especially in the parvicellular and magnocellular divisions, as well as the amygdalohippocampal area, similar to individuals with bipolar disorder, but which had normal pDyn levels in the magnocellular division [55].

### 2.2 Circuit 2 VTA Plus Nucleus Accumbens

#### 2.2.1 VTA

##### 2.2.1.1 In Rodents

There are 4 major subnuclei in the VTA: parabrachial pigmented nucleus, paranigral nucleus, parainterfascicular nucleus and interfascicular nucleus.

The paranigral nucleus and parabrachial pigmented area are zones rich in dopaminergic neurons while the parafasciculus retroflexus area and the tail of ventral tegmental area (tVTA) contain few dopaminergic neurons and are rich in neurons involved in modulating the activity of dopaminergic neurons [56].

In animal models of depression dysregulation in VTA was observed. The most intriguing fact is that in different paradigms there are different alterations in VTA neuronal behaviour. In learned helplessness and in chronic mild stress models the overall activity recorded in dopaminergic neurons of rodents was decreased, while in the chronic social defeat model tonic and phasic activity of dopaminergic neurons projecting from VTA towards NAc was increased [57]. These mechanisms imply that there are different subpopulations of dopaminergic neurons with different behaviours, afferents and efferents, leading to a complex mechanism of stressor processing and presenting the possibility of existing a large array of maladaptive mechanisms appearing in the case of depression. For example, in the chronic social defeat model, the activity of cholinergic neurons from laterodorsaltegmentum (LDT) is increased leading directly or indirectly through glutamatergic neurons to increased activity of dopaminergic neurons from VTA [58].

##### 2.2.1.2 In Humans

The ventral tegmental area (VTA) is a heterogeneous midbrain region composed of dopaminergic, GABAergic and glutamatergic neurons (see Figure 2.) with the dopaminergic neurons being the best understood. These dopaminergic neurons project to the medial and lateral shell of NAc, PFC (more specifically towards PV+ PFC interneurons), Amg and the HPC, the hypothalamus, the lateral habenula (LHb) and bed nucleus of stria terminalis (BNST) [59, 60]. On the other hand, VTA receives GABAergic afferents from the NAc and glutamatergic afferents from pyramidal neurons of PFC. From the lateral habenula, glutamatergic projections are sent through tVTA, which in turn sends GABAergic neurons of VTA [56, 60, 61]. The cholinergic afferents are present from LDT to VTA [62].

**FIGURE 2:**
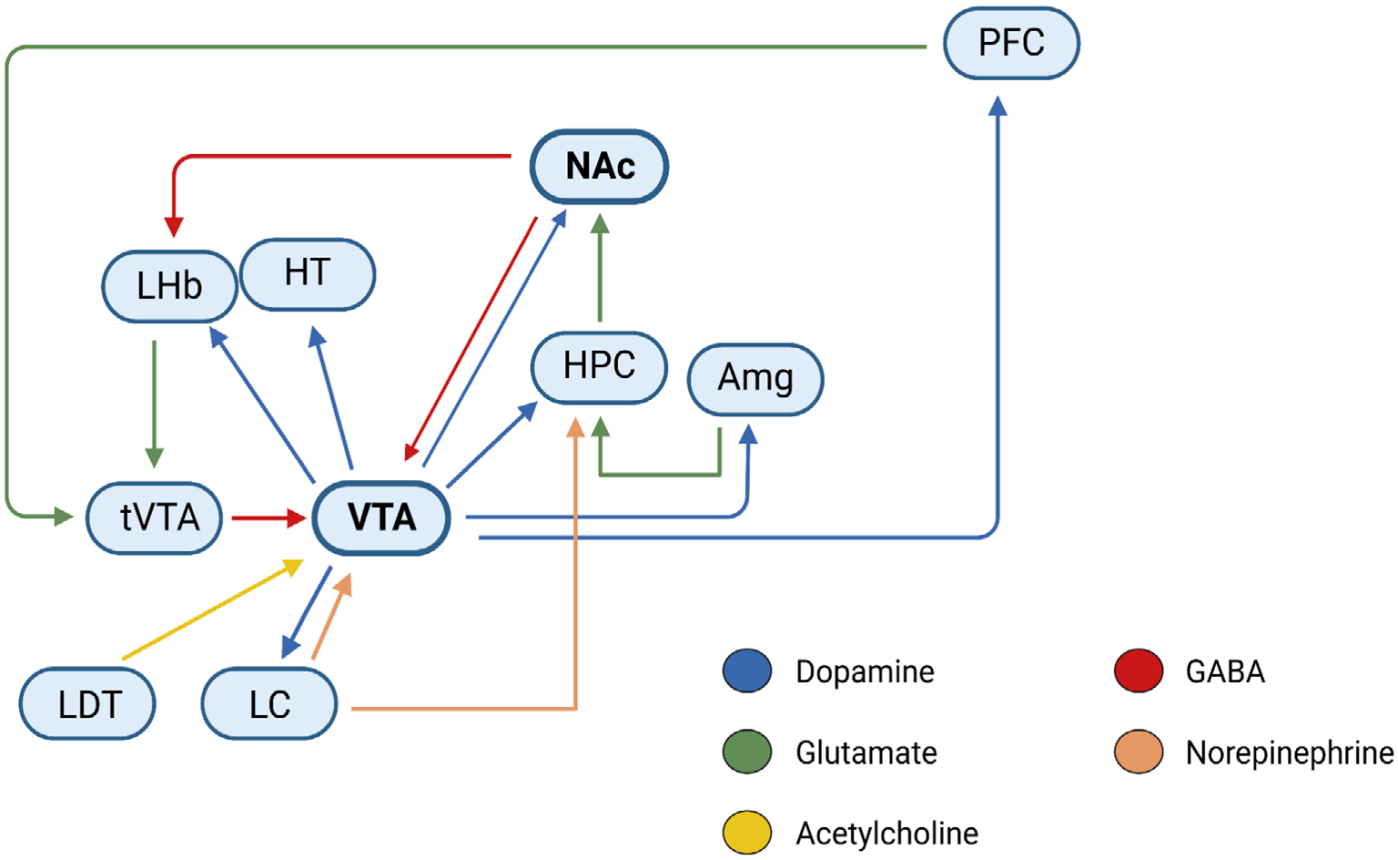
Representation of neural circuits and the neurotransmitters relevant for reward and motivation. Amg, amygdala; LDT, laterodorsal tegmentum; LC, locus coeruleus; LHb, lateral habenula; HT, hypothalamus; HPC, hippocampus; PFC, prefrontal cortex; VTA, ventral tegmental area; tVTA, tail of ventral tegmental area; Nac, nucleus accumbens; created in BioRender. Ciotei, C. (2026) https://BioRender.com/2uxqbcy.

In man, the VTA is a complex structure having neurons that activate during positive stimuli but also during stressors, assessing positive or negative values to stimuli. It is important in memory‐formation and learning as well as in motivation towards behaviours geared towards reward‐seeking or stressor‐avoidance. Considering some of the features of depressed individuals such as anhedonia (defined as the markedly diminished capacity to experience pleasure—specifically, a reduced interest or pleasure in activities that were previously found rewarding) and aboulia (refers to a lack of will, drive, or initiative for action, speech and thought), it becomes apparent that the VTA should play an important part in depression [63, 64].

#### 2.2.2 Nucleus Accumbens

##### 2.2.2.1 In Rodents

NAc is a structure that integrates information coming from multiple brain regions, many of them involved in depression [65]. Stress exposure seems to affect the behaviour of both D1‐medium spiny neurons (D1‐MSN) and D2‐MSN,underlying this region's relevance for depression. A decrease in dopaminergic transmission in the NAc is associated with depression in animal models [65].

##### 2.2.2.2 In Humans

NAc integrates information coming from multiple brain regions, such as glutamatergic projections from the PFC, HPC and BLA, dopaminergic projections coming from the VTA, NAc being one of the main targets of VTA dopaminergic projections [65]. There are 2 important interneuron populations at the level of NAc: medium spiny neurons (MSN), which are GABAergic in nature, and cholinergic interneurons. MSN are subdivided based on the type of dopamine receptor expressed in D1‐MSN and D2‐MSN [65]. D1‐MSN are predominantly involved in processing rewards, projecting back to VTA and to other regions such as the lateral habenula and the ventral pallidum. D2‐MSNs process aversive stimuli. D1 and D2‐MSN also synapse locally among themselves, leading to reciprocal modulation. The two MSN subpopulations also differ regarding the type of opioid peptide expressed: dynorphine is expressed in D1‐MSN, while D2‐MSN express enkephalins; D1‐MSN exerts its effects by activating KOR while D2‐MSN does so through DOR [66].

Deep Brain Stimulation of NAc seems to be a promising method of efficiently treating treatment‐resistant depression, further highlighting the importance of this structure in the neuropathophysiology of depression [67, 68], decreasing anhedonia and in some cases displaying an anxiolytic effect. A study conducted in a small number of institutionalized children showed that early life stress is associated with increased incidence in depression that correlates with hypoactivity and less reactivity of NAc [69].

## 3 Kappa‐Opioid Receptor/Dynorphines (KOR/Dyn Opioid) System

### 3.1 Circuit 1: Hippocampus‐Amygdala‐Prefrontal Cortex

#### 3.1.1 Pfc

##### 3.1.1.1 In Rodents

There are pDyn expressing neurons at the level of IL in either somatostatin‐positive (SST+) GABAergic interneurons or glutamatergic neurons [70]. It is interesting to note that the rodent mPFC has a higher proportion of SST+ neurons compared to parvalbumine‐positive (PV+) neurons [24], suggesting that the KOR/Dyn system plays an important role in PFC‐mediated rodent behaviour.

KOR‐stimulation at the level of PL leads to place aversion [71]. Systemic or intra‐mPFC administration of a KOR agonist leads to the cessation of BLA‐evoked potentials at the level of mPFC [72]. BLA neurons act on mPFC through KOR/Dyn system [27, 72]. While KOR activation might lead to an inhibition of BLA‐projecting neurons, it can also influence the behaviour by its action on other circuits, especially those involving interneurons. Studies performed by Yarur et al. 2023 [73] suggest that in the mPFC KOR/Dyn acts as a switch to increase the importance of certain pathways by inhibiting signals coming from KOR‐positive neurons. In the same time, it can increase activity in other circuits by inhibiting SST and PV+ interneurons as well as polysynaptic inhibition dependent upon SST‐IN, leading to a net effect of favouring signals from KOR‐negative projections such as the ventral hippocampus.

The ventral hippocampus, apart from mediating anxious behaviour [42], is also important in fear expression as well as fear extinction in conditioned‐fear [30]. These aspects are intriguing, since at the level of IL footshocks or threat‐signals (signals that were associated with footshocks) lead to rapid activation of dyn‐neurons which caused activation of certain neural networks associated with vigilance and decreased freezing [70]. These neurons co‐express either GABA or glutamate with Dyn, hence the effect of these neurons could be quite different from the pharmacological activation or inhibition of KOR.

##### 3.1.1.2 In Humans/Primates

Apart from the affective elements, depression also is associated with impairment of cognitive functions such as decreased processing speed, working memory and attention deficits [74, 75, 76]. The particular aspect of these symptoms is complex. For example, working memory deficits are observed in depressed human individuals generally when the tasks used don't constrain the attention of the participant through a thorough‐enough set of instructions but rather leave it to be more self‐regulated [75]. Depressed patients tend to have poorer outcomes in tests evaluating for the ability to direct, sustain or divide attention [74], and this in turn can influence perception and decision making. Particularly, a decreased ability to select the object of one's interest can explain the poor results in working memory tests and might explain the tendency of depressed individuals to spend more time over negative‐valence stimuli compared to neutral or positive ones [77]. A depressed individual may need to recruit greater prefrontal cortex activation than a healthy control subject in order to achieve comparable task performance [78]. In other words, individuals suffering from MDD might need to allocate more mental resources for simpler tasks than non‐depressed individuals.

The KOR/Dyn system seems to be involved especially in the effects of stress over working memory. In Rhesus macaques, systemic KOR‐antagonist—navacaprant or NMRA‐140 administration protects against working memory impairments produced by a pharmacological‐induced stress state [79]. The authors assumed that the effect is explained by the antagonist interfering with KOR‐modulated release of norepinephrine (NE) and dopamine (DA) from LC and VTA, an effect brought about by KOR inhibiting inhibitory neurons at the level of CeA which in turn leads to release of NE and DA at the level of PFC. This is certainly not the only way in which KOR can influence working memory since KOR activation at the level of mPFC can lead to working memory impairment and the effects of systemic KOR agonist administration are alleviated by mPFC administration of nor‐binaltrophimine (nor‐BNI) [80].

#### 3.1.2 Hippocampus

While greatly expressed in the hippocampus, KORs show variations between species regarding distribution and affinity [81] with DG presenting the highest density of dynorphins and KORs [82]. Dynorphins are found in the dendrites and axons of granule cells of DG. Exposure to stressful events increases hippocampal dynorphin expression. Sudden increases in dynorphin levels are associated with decreased synaptic transmission and with prolonged inhibition of the potentiation of cell‐perforant path and mossy fibre synapses [83].

##### 3.1.2.1 In Rodents

The Wistar‐Kyoto rat, which is a model for depression, is reported to have lower levels of brain‐derived neurotrophic factor (BDNF) in PFC and in the hippocampus compared to standard Wistar rat [84]. In the brain of Wistar‐Kyoto rats, the highest pDyn‐coding mRNA was reported to be found in the DG [85].

Indeed, in a learned helplessness paradigm, CA3 injection of nor‐BNI produced antidepressant effects [86]. In male Sprague–Dawley rats, intracerebroventricular administration of nor‐BNI increased BDNF mRNA expression in the hippocampus, but only on the first day after administration [87]. BDNF is the most expressed neurotrophin in mature brains, involved in neuroprotection, brain development and synaptic plasticity. BDNF mRNA levels have been shown to increase after a few days of antidepressant treatment [88]. On the other hand, administration of the KOR agonist U50488 has been shown to notably decrease BDNF expression in the hippocampus and therefore elevate symptoms of depression in mice [89]. Imipramine, fluoxetine and citalopram are able to counteract U50488‐induced BDNF reduction in the hippocampus but not in the PFC [90].

#### 3.1.3 Amygdala

##### 3.1.3.1 In Rodents

KOR expression is upregulated in the BLA by fear‐conditioning. Fear‐extinction leads to a decrease in KOR expression, showing the importance of KOR/Dyn in fear memory [91]. Furthermore, BLA and CeA infusion of the KOR antagonist JDTic reduced fear expression, while BLA‐only infusion had anxiolytic effects [91].

U‐50488 increases activity of BLA pyramidal neurons in a MAPK‐pathway dependent manner, a process which is reversed by either antagonism of KOR, using nor‐BNI or by MAPK‐pathway antagonism [92]. Probably this effect is mediated by KOR inhibiting inhibitory interneurons at the level of BLA. KORs present in BLA neurons also act as modulators for downstream circuits. KORs are present on the terminals of a set of efferents emerging from BLA to NAc and negatively modulate glutamatergic output to D1‐MSN [93]. Similarly, presynaptic KOR inhibits glutamatergic BLA neurons at mPFC level, the effect being blocked by nor‐BNI administration [71].

The BLA‐excitatory neurons project to the vHPC, which is an important circuit in orchestrating anxiety‐related behaviour, the activation of this pathway being anxiogenic while its inhibition produces anxiolysis [94]. In opiate withdrawal, these neurons are expressing an increased number of KORs. Silencing of their expression in this pathway alleviates anxiety‐like behaviours associated with morphine withdrawal. Supplementary, chemogenetically inhibiting this circuit and systemic administration of nor‐BNI proved to have a synergistic effect in limiting anxious manifestations [95]. One interesting question is why hyper‐expression of KORs, an inhibitory receptor, could lead to the stimulation of these neurons. It is known that sometimes KORs can be coupled to Gs proteins [18] and some research reported the ability of some KORs, in only certain VTA neurons, to change from inhibitory to excitatory under acute stress or CRF exposure [96]. While this underlines the complex character of KOR behaviour and the possibility of switching its action, there are numerous differences to the morphine‐withdrawn mice; for example, the acute compared to a more chronic context, the location VTA compared to BLA neurons and the overall conditions of the experiments [96].

The amygdala also plays a role in modulating the stress‐axis as intra‐BLA pharmacological blockade of KORs (using nor‐BNI or JDTic) counteracts the anxiogenic effects of CRF intracerebroventricular injection in mice [97] and in rats [91]. Bruchas et al. 2009 showed that the anxiogenic effects of CRF are in part caused by a corticotropin ‐releasing factor type 1 ‐receptor (CRF 1‐R)‐dependant activation of the KOR/Dyn framework at BLA level [97].

A subset of inhibitory CeA neurons are also CRF‐producing, releasing apart from GABA, an ensemble of other neuromodulatory peptides such as CRF, Dyn and neurotensin. Under basal conditions, only GABA is released from these neurons, and it plays a role in keeping in check basal‐anxiety; suppression of GABA release in this case leads to increased anxiety. Under aversive conditions, CRF, Dyn and neurotensin are also released, with CRF and Dyn increasing anxiety and being involved in fear conditioning, while neurotensin plays a more counterbalancing role, suppression of only neurotensin synthesis in these neurons increasing cued‐elicited fear‐behaviour [98].

CeA also mediates pain‐evoked behaviour, mostly by processing the emotional valence of pain rather than the actual sensory aspect of it. By inhibiting the inhibitory neurons that suppress CRF‐producing neurons in CeA, KORs activation mediates multiple negative effects, leading to increased anxiety, place aversion and increased vocalization in noxious‐stimuli naive mice [99].

Although the amygdala is seen more canonically as a fear centre, it is also involved in addiction and substance abuse, especially when stress is overlapping. For example, mice exposed to stressful conditions (repeated forced swim tests, FST) consumed larger quantities of alcohol in a chronic intermittent ethanol exposure paradigm compared to control non‐stressed mice. Microinfusions of nor‐BNI into CeA or into BNST led to a reduction in alcohol consumption [100]. This is important because often psychiatric diseases (such as MDD or anxiety disorders) predispose to drug consumption, including alcohol abuse.

Nor‐BNI administration was able to prevent development of social avoidance in male mice exposed to 14 days of social defeat stress. Neural activity quantification by c‐fos staining reveals that the amygdala is involved in the development of social avoidance behaviour [101]. Increased signalling in the ERK pathway, which is a well known molecule engaged in the KORs transduction cascade [102] and mood regulation [103], and elevated expression of dynorphin in the amygdala but not in other structures of the limbic system suggest the involvement of KOR/Dyn in mediating social avoidance after chronic social defeat [101].

##### 3.1.3.2 In Humans

Postmortem molecular evidence found that reduced pDyn mRNA expression in the periamygdaloid cortex of postmortem human brains from both heroin users and individuals with major depressive disorder—a shared molecular signature linking addiction and depression through this same circuit, with prodynorphin reduction also noted in amygdalohippocampal/accessory basal nuclei of MDD and bipolar disorder subjects [104].

### 3.2 Circuit 2: Ventral Tegmental Area—Nucleus Accumbens

#### 3.2.1 VTA

##### 3.2.1.1 In Rodents

KOR seems to be localized presynaptically in dopaminergic VTA‐neurons [71], the majority of excitatory glutamatergic neurons being projected to the PFC are KOR‐negative, except for some neurons sending information from BLA, thalamus and the contralateral PFC and on local inhibitory interneurons [73].

KOR is involved in different mechanisms responsible for modulation of DA neurons of VTA. It was observed that an increase in activity of a dopaminergic neuron from VTA will lead to an increase in NO production that will cross the neuronal membrane and increase the release of GABA from nearby interneurons. When exposed to an even acute stressor this mechanism that keeps DA neurons in check is altered through the activation of presynaptic KORs leading to a constitutive activation of this receptor and to the inhibition of inhibitory GABAergic neurons and respectively to increased firing rates in VTA's dopaminergic neurons. This phenomenon is reversed by nor‐BNI. Competitive KOR antagonists can prevent this effect if administered before the stressful stimulus [105]. A knockout murine model in which KOR were specifically eliminated from dopaminergic neurons of VTA showed a propensity towards less anxiety in certain behavioural tests such as the open field test and the light/dark box but in other tests such as the elevated plus maze no effect was observed.

#### 3.2.2 Nucleus Accumbens

##### 3.2.2.1 In Rodents

It was reported that in depression models such as learned helplessness and tests evaluating depressive behaviour such as the FST, administration of a KOR antagonist directly at the level of NAc was associated with alleviation of depressive behaviour [11, 106].

Similar effects were observed by inhibiting cAMP response element‐binding protein (CREB) at the level of NAc where it is responsible for upregulating the expression of pDyn. In a rodent model of depression induced by morphine withdrawal, depressive behaviours appear after 4 weeks of abstinence and are associated with increased KOR and pDyn expression in NAc. Intra‐NAc injection of microdoses of KOR antagonists exhibited antidepressant effects while a single dose administered intra‐peritoneally had temporary antidepressant effects. Chronic administration of KOR antagonists had prolonged effects, up to 2 weeks [107, 108]. Similar results of antidepressant effects were reported for K.O mice lacking NAc KORs [107]. In morphine‐withdrawal induced models of depression as in other models of depression, CREB involvement is stipulated, influencing the expression of pDyn and BDNF [107].

## 4 Limitations

Several limitations should be considered when interpreting the evidence reviewed here. Most of the studies of the KOR/Dyn system are based on animal models, particularly rodents and frequently do not adequately account for biological variables such as sex, age, and social status. In addition, translation to human depression and anxiety remains limited by the methodological constraints of currently available neuroimaging approaches.

### 4.1 Sex‐Related Differences in Preclinical Studies

Sex is an important but often insufficiently addressed biological variable. This is particularly relevant because depression is more prevalent in females, with differences emerging as early as adolescence [109], and because the estrous cycle can modulate mesolimbic circuitry and reward‐related behaviour in female mice [110]. Sex‐dependent differences in KOR localization have also been reported in rodents and humans [111, 112].

These differences may reflect both hormonal and chromosome‐related mechanisms. Oestrogen signalling can regulate KOR/MOR heterodimer formation and modify behavioural responses to KOR agonists [113, 114], while the KOR/Dyn system itself contributes to oestrogen‐mediated regulation of luteinizing hormone release [115]. In addition, female mice show reduced KOR‐mediated analgesia compared with male mice independently of circulating sex hormones [116], and female rats appear less sensitive to KOR‐dependent attenuation of reward‐related signalling through mechanisms that are not entirely hormone‐dependent [117]. Thus, failure to account for sex‐related biological variability may lead to incomplete conclusions regarding KOR/Dyn function.

### 4.2 Non‐Sex‐Related Differences in Preclinical Studies

Age is another important source of variability. KOR activation produces weaker dysphoric effects in adolescent than in adult rats [118], and anxiolytic‐like effects have been reported in adolescent rodents following systemic KOR stimulation [119]. Age‐dependent circuit effects have also been observed, including differential modulation of GABA release in the basolateral and central amygdala [120], despite similar KOR mRNA expression across age groups. These findings suggest developmental differences in downstream signalling or circuit responsiveness.

Social context may further influence KOR/Dyn function. In non‐human primates, KOR availability differs according to social status, including dominant versus subordinate position [121]. This variable may be particularly relevant in studies involving social stress, defeat, isolation, or hierarchical interactions.

### 4.3 Limitations of Human Studies

The strong reliance on animal models limits direct translation to psychiatric disorders, which include subjective and cognitive experiences that cannot be fully reproduced experimentally. Human studies therefore depend largely on fMRI and PET imaging.

fMRI is affected by substantial inter‐ and intra‐individual variability and by physiological and environmental factors, complicating standardization and interpretation [122]. It also provides only indirect measures of neuronal activity and cannot identify the specific neurotransmitter systems involved.

PET studies using high‐affinity KOR radiotracers have been applied to depression and addiction [123, 124, 125], but are limited by tracer kinetics, blood–brain barrier penetration, cost, methodological complexity, radiation exposure, relatively small sample sizes, and restricted temporal and spatial resolution.

These limitations explain the continued importance of animal‐based approaches such as optogenetics, chemogenetics and electrophysiological recordings, which allow direct circuit manipulation with high temporal and spatial precision. However, their mechanistic advantages should be balanced against their limited ability to reproduce the complexity of human affective disorders.

## 5 Conclusion

Understanding the complex nature of the KOR/Dyn system, while inherently challenging, is essential for developing new drugs capable of improving the management not only of MDD and AD but also of addressing frequently associated conditions such as substance abuse. It is worth noting that variables such as age, sex, and social hierarchy rank can influence experimental outcomes, and future investigations into KOR/Dyn system function should account for these factors when designing experimental protocols.

## Author Contributions

Conceptualization, C.C., H.P., C.L. and O.A.C.; methodology, C.C., H.P. and C.L.; resources, C.L. and M.C.; writing – original draft preparation, C.C., A.‐A.M., M.F.T., C.L. and H.P. and.; writing – review and editing, C.C., H.P., C.L., O.A.C. and R.‐C.M.; visualization, C.C.; supervision, I.F., O.A.C., H.P. and M.C.; project administration, H.P., R.‐C.M., I.F., and O.A.C. All authors have read and agreed to the published version of the manuscript.

## Funding

The authors have nothing to report.

## Conflicts of Interest

The authors declare no conflicts of interest.

## Data Availability

Data sharing not applicable to this article as no datasets were generated or analysed during the current study.
